# Women with Moustaches

**Published:** 1881-07

**Authors:** 


### WOMEN WITH MOUSTACHES.

When you see a dark woman with large, bushy eyebrows, which nearly grow together, you may be pretty sure that she can grow an imperial if she will only take the necessary pains. Hirsute adornments are not so frequent in young women, although they sometimes occur. A girl often has a heavy growth of down upon the upper lip or the chin. It annoys her, and she keeps feeling it and pulling it continually. Perhaps she endeavors to clip it with a scissors, or, in some cases, to shave it away. The result is a heavier growth next time, which becomes so prominent that it must be removed.

The most frequent place where the hair makes its appearance is on the upper lip or the chin, although it sometimes appears on the side of the face, and even on the throat. As a general thing, these bearded women which are advertised by shows are frauds, but more than one woman could raise a heavy beard if she only wishes to cultivate it—a great many more women than is generally supposed. They hide the traces that the shaving leaves with heavy doses of powder and plaster. Whenever you see a lady, especially if she be dark-featured, wearing a heavy coating of lily-white, one of the conclusions is that she has been shaving, although it is not the only inference. One thing is certain, a lady who shaves must use powder in large quantities, and there are many ladies who shave.

All the books recommend depilatories, but these are usually of little service. They are composed mainly of quick lime and opient, which is a preparation of arsenic ; and the only effect is to cut off the hair to the surface of the skin, leaving the root intact to grow again. In what is known as the electric method, the patient sits in a chair, holding in her hand a sponge which is connected with the negative pole of an electric battery. The operator holds a needle, or fine wire, which is connected with the positive pole, and this he thrusts, with a quick motion, down into each cell or follicle, thus destroying the root. Every time the needle touches the skin a severe shock is caused, which will cause a nervous patient to cry out with pain ; and if the operator is a bungler, or if his nerve is not very steady, he will miss his mark, and cause more intense agony. Not more than one, or, at most, two dozen hairs can be attended to at one sitting.

Sometimes women attempt to operate upon themselves. They heat needles and endeavor to introduce them into the capillary cells. The usual result is that the carbon, which accumulates in the needle during the heating, is imbedded in the epidermis, or underskin, and a first-class case of tattooing is the result. Then, again, many ladies use acid for the purpose, and permanently scar their faces. Others remove the hair with tweezers.

---

LIFE being short, and the quiet hours of it few, we ought to waste none of them in reading valueless books, and valuable books should, in a civilized country, be within the reach of every one.

### SANITARY DEPARTMENT OF "HALL'S JOURNAL OF HEALTH."

We are daily receiving requests from our readers to purchase for them "HEALTH FOODS," "FOOD MEDICINES," PURE BAKING POWDERS, and various MEDICINES that our experience has taught us the value of ; also standard books ; and last but not least, many families rely on us to procure for them PURE BOVINE VACCINE MATTER, which is the only vaccine matter that is always safe. These commissions already keep one person employed much of the time. We have therefore determined to make this a branch of our regular business, and announce ourselves ready to fill orders for GLUTEN of WHEAT, GLUTEN COFFEE, COOKED FOODS, as CRUSHED WHEAT, BARLEY, OATS, CORN and RYE, also the various preparations of MALT, and for books, medicines, vaccine matter and any other like articles that can be found in New York market.

E. H. GIBBS & CO.

BARLEY WATER.—I have found this useful in private practice to keep up the strength of a patient: A cupful of barley in two quarts of water, allowed to boil two hours. About an hour after it is on the fire, add a dozen stoned raisins ; strain before serving. If I wish a stimulant, I put in the glass or cup in which I serve it a teaspoonful of brandy or sherry wine. It ought to be cooked in a porcelain-lined vessel or it is apt to discolor.

HOW TO MAKE YOURSELF UNHAPPY.—In the first place, if you want to make yourself miserable, be selfish. Think all the time of yourself and your things. Don't care about anything else. Have no feelings for any but yourself. Never think of enjoying the satisfaction of seeing others happy ; but rather, if you see a smiling face, be jealous lest another should enjoy what you have not. Envy every one who is better off than yourself ; think unkindly toward them, and speak lightly of them. Be constantly afraid lest some one should encroach on your rights ; be watchful against it, and if any should come near youı things snap at them like a mad dog. Contend earnestly for everything that is your own, that may not be worth a pin. Never yield a point. Be very sensitive, and take everything that is said to you in playfulness in the most serious manner. Be jealous of your friends lest they should not think enough of you ; and if at any time they should seem to neglect you, put the worst construction upon their conduct.

### LIFE INSURANCE WITHIN THE REACH OF ALL.

INSURING one's life, which most people deem a duty, has hitherto been a luxury too costly for people of moderate means. Life insurance companies, in order to accumulate their enormous surplus funds, in many cases amounting to millions of dollars, have fixed their rates of premiums at figures which, we repeat, are prohibitory.

The extravagance of what we may properly call this old system of insurance has driven great numbers of persons to the adoption of a newer and better system of life insurance, within the reach of those to whom it is of the greatest use, to wit, persons of moderate means. This system is strictly mutual, and therefore the safest of any; since, after providing for the ordinary expenses of economical management, members are only taxed the small sums required from time to time to make up the losses by death; thus members are not taxed three or four times as much as is necessary in order to establish an enormous surplus fund in which they have no interest beyond the amount of their insurance. We believe in the new plan of life insurance, and have shown our faith by taking policies of insurance in two associations. We will forward all necessary printed information to any reader of the JOURNAL who will take the trouble to write to us on the subject.

**KNIGHT BROTHERS,**

SOLICITORS OF

UNITED STATES AND FOREIGN

**PATENTS,**

**Counsellors in Patent Causes**

AND

MECHANICAL EXPERTS,

AGENCIES AT PRINCIPAL FOREIGN CAPITALS.

**No. 633 F Street, N. W., Washington, D. C.;**
**75 West Fourth Street, Cincinnati, Ohio;**
**817 Olive Street, St. Louis, Mo.**

**SEND FOR BOOK OF INSTRUCTIONS.**

### DR. HALL'S PORTABLE SPIROMETER.

NO PHYSICIAN questions the value of the spirometer for exercising and expanding the lungs, and for measuring their capacity. The great difficulty has been to find an instrument that can be compressed into a small compass when not in use, so that the physician or patient may conveniently carry it from place to place as circumstances require. Another obstacle to the more general use of this truly valuable instrument has been its price. The unwieldy spirometer of a few years ago cost from $10 to $20. Dr. Hall's Portable Spirometer sells for $7.50, and will be sent free to any address on receipt of the price by the editor of this journal.

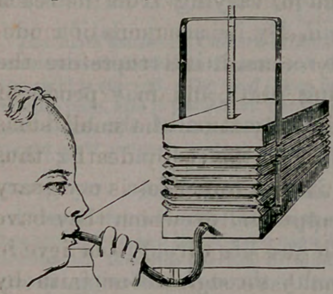

It is 6 by 12 inches, and when closed it stands only 3 inches high, and weighs less than 3 pounds.

This cut represents Dr. Hall's Portable Spirometer, partly inflated, in order to show the manner of using it. When not in use it can be closed together so as to occupy the small space above stated. The scale and guide are hinged, so that they close down on the top of the spirometer, and occupy but little space. The top is of polished black walnut. The brass-work is plated with silver or nickel.

HOW TO USE THE SPIROMETER.

The instrument should be used only in a perfectly ventilated room, which is entirely free from dust, or else in the open air, away from the dust and smoke and bad odors of the streets of a city. When it is remembered that the capacity of the lungs of a man of medium size is 150 to 250 cubic inches, and that in the act of breathing naturally, or rather as some have acquired the habit of breathing—which is far from natural—the lungs take in only from 20 to 40 cubic inches of air at each inspiration, leaving from 130 to 230 cubic inches of air cells almost always in a dormant condition, the importance of regular and systematic exercise for the lungs will be appreciated. But, like all good things, this sort of lung exercise must be indulged in moderation. Draw a full breath and then blow through the rubber tube into the spirometer; at the same time watch the figures on the scale as it rises. The figures seen on the scale as it passes through the top of the guide indicate the number of cubic inches of air blown into the spirometer.

It is now very generally conceded that mankind start quite evenly on the journey of life, in spite of supposed hereditary tendencies to pulmonary consumption, and that with proper care the average term of life is not shortened by these hereditary tend n ies. This being the case, it is far better to enjoy health and long life through means wh ch the naturally robust are not compelled to resort to, than to believe in or lament over the inheritance of ills that do not exis . We believe for all persons who are confined much of the time in doo s, particularly in cases where there is a tendency to weak lungs, the regular use of the spirometer in the open air is highly beneficial. Its use just before bedtime is said to induce sleep, by drawing blood from the brain to the lungs.

### CONSTIPATION.

CONSTIPATION becomes alarmingly frequent as people acquire the means of living luxuriously without regular manual labor. It is still more frequent among persons who become so through ignorance of the penalties that follow a want of attention to the regular demands of nature.

When fecal matter is retained in the rectum more than twenty-four hours, it commences its destructive work, notably in two ways. *First*, It is absorbed into the system, and acts as a blood poison. One of the worst results from blood poisoning from constipation is to cause disease of the mind, varying from habitual moroseness to actual insanity. *Second*, By its accumulation and impacting in the lower portion of the rectum it interrupts the circulation of the blood in the adjoining parts, and may produce, from this cause alone, piles, fistula, inflammation of the bladder, disease of the prostate gland, or cancer of the rectum. In fact, most of these diseases are developed in this way. To a very great extent we hold our health and happiness, even our lives, in our own hands. If we sacrifice health and life through our ignorance, or indifference, we pay the penalty through suffering, more severe because more prolonged than instant destruction. From whatever cause, there is, unfortunately, great ignorance in regard to the laws of health among a class of people who are otherwise intelligent. How many parents are there who look carefully to the habits of their children, upon which all that will make life desirable for them so much depends? Cathartics and clysters should not be used in constipation. They increase the trouble.

That which has, perhaps, met with most general favor relates to the diet. Certain foods have been regarded as specifics for this complaint, and particularly "Graham bread." This often induces activity of the bowels, but it ruins digestion like other cathartics, in a manner which we have heretofore fully explained in the pages of this journal. Kneading the bowels has sometimes afforded relief, but it affords no permanent benefit, as a rule. We have, however, found a remedy for constipation and for piles which is always safe, and is attended with no inconvenience or discomfort. We have prescribed it in hundreds of cases. It has usually afforded prompt relief, and many patients who have suffered for years believe that they have been permanently cured of constipation by its use. This remedy is "Nelaton's Suppository." It is prepared and sold by Hall & Ruckel, wholesale druggists, 218 Greenwich street, New York, at 50 cents and $1.00 a box, and is sent free by mail on receipt of price.

